# Towards breath sensors that are self-powered by design

**DOI:** 10.1098/rsos.220895

**Published:** 2022-09-21

**Authors:** Lucy Fitzgerald, Luis Lopez Ruiz, Joe Zhu, John Lach, Daniel Quinn

**Affiliations:** ^1^ Mechanical and Aerospace Engineering, University of Virginia, Charlottesville, VA, USA; ^2^ Electrical and Computer Engineering, University of Virginia, Charlottesville, VA, USA; ^3^ Electrical and Computer Engineering, George Washington University, Washington, DC, USA

**Keywords:** piezoelectricity, energy harvesting, flow sensing, implantables, body sensors, self-powered

## Abstract

Piezoelectric materials are widely used to generate electric charge from mechanical deformation or vice versa. These strategies are increasingly common in implantable medical devices, where sensing must be done on small scales. In the case of a flow rate sensor, a sensor’s energy harvesting rate could be mapped to that flow rate, making it ‘self-powered by design (SPD)’. Prior fluids-based SPD work has focused on turbulence-driven resonance and has been largely empirical. Here, we explore the possibility of sub-resonant SPD flow sensing in a human airway. We present a physical model of piezoelectric sensing/harvesting in the airway, which we validated with a benchtop experiment. Our work offers a model-based roadmap for implantable SPD sensing solutions. We also use the model to theorize a new form of SPD sensing that can detect broadband flow information.

## 1. Introduction

Piezoelectric devices have wide-ranging sensing/harvesting applications. These devices are typically used in either a sensing capacity, by generating an electric output that correlates to deformation [1–3], or in a harvesting capacity, by using that output to charge a storage element [4–8]. The circumstances that warrant these uses are different enough that the trade-offs between sensing and harvesting usually preclude simultaneous sensing and harvesting by one device.

In special cases, the sensing and harvesting functionality can overlap: if the harvesting rate scales with a variable of interest (e.g. flow rate), then the duty cycle at which the system operates is itself a proxy for the variable of interest, and the sensor is ‘self-powered by design (SPD)’. A piezoelectric energy harvester on a fish fin, for example, can act as a SPD biotag [9]. When the fish is swimming, its harvester pings a receiver, and thus the harvesting rate is a proxy for the fish’s activity.

Here, we present an exploration of SPD sensing as a way of measuring human breaths from within the airway. Our intent was not to create a viable airway sensor, but rather to validate a framework for designing an airway sensor in the future. Implantable airway sensors could revolutionize the lifestyles of patients with a chronic respiratory disease. Unlike existing sensing solutions, such as nasal cannulae or chest straps [10–16], an implantable airway sensor could offer continuous, long-term data collection without the stigma or inconvenience of a wearable device. Implantable sensors must overcome the ‘nightmare of battery replacement and disposal’ [17], so they are prime candidates for minimalist, batteryless, solutions like SPD sensing.

SPD sensing has thus far been explored for piezoelectric structures driven to resonance by a broadband turbulent flow. The concept has been demoed for measuring deformation in fluttering flags [18], activity in swimming fish [9], flow speeds in wind [19], flow speeds in water faucets [20] and flow speeds in forced exhalations [21]. Designing for resonance is effective, because resonance maximizes the efficiency of a harvester [7,8]. By contrast, a SPD sensor driven by slow breaths may be forced well below its resonant frequency. This condition is considerably less efficient [22], but if combined with duty-cycled and/or ultra low-power electronics, it may still enable a feasible SPD airway sensor.

To explore the feasibility of self-powered breath sensing, we propose a model for piezoelectric films/cantilevers (henceforth ‘piezofilms’) driven at the sub-resonant frequencies of human breaths (frequencies approx. 0−90 breaths per minute (BPM), amplitudes approx. 0−7 l). Our model combines elements of fluid dynamics, elasticity theory, piezoelectricity and circuit design. To test our model, we built a transient airflow generator that actuates a rectangular polyvinylidene difluoride (PVDF) beam at frequencies and amplitudes typical of human breaths. Our results offer a roadmap for self-powered sensors that measure human breath, but also for those that measure low-speed oscillating flows more generally. Model-driven self-powered sensors could potentially be used to diagnose or monitor a variety of flow-related diseases in the body.

## 2. Modelling

### 2.1. Elastic beam theory governs sensor deflections

Several studies have modelled piezoelectric energy harvesters by combining ideas about aeroelastic coupling with electromechanical coupling to create a single set of equations (e.g. [2,7,8,22]). What differentiates our model is that it is dimensionless and incorporates a user-defined circuit impedance—features designed to make our model generalizable to other SPD systems, including those operating far from resonance. We modelled a piezofilm as a constant-property elastic beam under uniform harmonic forcing. Under these assumptions, the beam’s displacement is governed by linear beam theory [23]

2.1 $$\frac{ewh^{3}}{12}\frac{\partial^{4}\delta }{\partial x^{4}}=-m\frac{\partial^{2}\delta }{\partial t^{2}}+qe^{j\omega t},$$

where *x* is distance along the beam, *t* is time, *q* is the magnitude of the forcing per unit length, *ω* is the angular frequency of the forcing, j is the square root of −1, and *δ*, *w*, *h*, *e* and *m* are the lateral displacement, width, thickness, elastic modulus and mass per length of the beam, respectively. We assumed that the beam’s cross-sectional area moment of inertia is that for a rectangle, *wh*^3^/12, and that the drag it experiences follows $q=\frac{1}{2}\rho C_{D}w\ell u^{2}$, where *ρ* is the density of air, *C*_D_ is the drag coefficient, and *u* is the incoming flow speed. For drag coefficient, we used a typical value for a flat plate perpendicular to the incoming flow (*C*_D_ = 1) [24].

Because our goal was to set up a variable-lean framework that may scale to other SPD systems, we non-dimensionalized our model using the following definitions:

2.2 $$X\equiv \frac{x}{\ell },\;T\equiv \omega t,\;\Delta \equiv \frac{\delta }{\ell },\;\lambda \equiv {\left(\frac{12m\omega^{2}\ell^{4}}{ewh^{3}}\right)}^{\frac{1}{4}}\mathrm{and}\;Q\equiv \frac{12q\ell^{3}}{ewh^{3}},$$

where ℓ is the beam’s length, which leads to an equation for dimensionless beam displacement

2.3 $$\frac{\partial^{4}\Delta }{\partial X^{4}}=-\lambda^{4}\frac{\partial^{2}\Delta }{\partial T^{2}}+Qe^{jT}.$$

The dimensionless groups *λ* and *Q* represent ratios of inertial and drag forces to elastic forces. Due to the complex forcing, the solution for dimensionless deflection is a complex phasor that can be evaluated by, for example, considering its real component. Assuming an oscillating solution and using the boundary conditions for a fixed-free cantilever (Δ(0, *T*) = Δ_*x*_(0, *T*) = Δ_*xx*_(1, *T*) = Δ_*xxx*_(1, *T*) = 0) leads to an exact solution for Δ

2.4 $$\Delta =\frac{Q}{\lambda^{4}}e^{jT}(c_{1}\sin(\lambda X)+c_{2}\cos(\lambda X)+c_{3}\sinh(\lambda X)+c_{4}\cosh(\lambda X)-1),$$

where the coefficients *c*_*n*_ are given in the electronic supplementary material. Based on linear theory, higher normalized loading (*Q*) causes higher normalized deflection (Δ) without bound. In reality, nonlinear stresses prevent the beam from deflecting more than distances of approximately *π*ℓ/2 (arc length of a quarter circle with radius ℓ). Anticipating large deflections, we used a modified deflection function that caps deflections at *π*ℓ/2 (i.e. caps Δ at *π*/2)

2.5 $$\Delta^{\prime }=\Delta \cdot \frac{\pi }{2}\cdot \frac{1-e^{-|\Delta (1,0)|}}{\left|\Delta (1,0)\right|}.$$

The modified dimensionless deflection (Δ′) converges to linear beam theory as *Q* → 0 and converges to π/2 as *Q* → ∞.

### 2.2. Piezoelectric equations govern circuit response

For a bimorph piezofilm, the average strain in the piezofilm is the strain halfway between the neutral axis and the beam’s surface, averaged over the length of the beam [25]

2.6 $${\bar{\epsilon }}_{xy}=\int_{0}^{1}\left(\frac{1}{2}\frac{h}{\ell }\frac{\partial^{2}\Delta^{\prime }}{\partial X^{2}}\right)dX=Q\hat{\lambda }\frac{h}{\ell }e^{jT},$$

where $\hat{\lambda }$ is a dimensionless ratio involving *λ* introduced for notational convenience: $\hat{\lambda }\equiv (\sin\lambda -\sinh\lambda )/(2\lambda^{3}(1+\cos\lambda \cosh\lambda ))$.

The voltage between the piezofilm’s electrodes (*v*_p_) can be modelled by the piezoelectric constitutive equations reformulated to include macroscopic properties like normalized current [26]

2.7 $$\frac{dV_{p}}{dT}=\frac{d{\bar{\epsilon }}_{xy}}{dT}-I,$$

using normalized variables

2.8 $$V_{p}\equiv \frac{v_{p}}{\gamma \ell /c_{p}}\;\mathrm{and}\;I\equiv \frac{i}{\gamma \omega \ell },$$

where *i* is the current induced by the piezofilm, *γ* is the generalized electromechanical coupling factor and *c*_p_ is the piezoelectric output capacitance (see electronic supplementary material for details). To be consistent, we similarly defined normalized impedance (*z*), resistance (*r*) and capacitance (*c*) as *Z* ≡ *zωc*_p_, *R* ≡ *rωc*_p_ and *C* ≡ *c*/*c*_p_. The impedance *z* is the impedance of the total circuit, i.e. the piezofilm and the external load.

Solving equation (2.7) requires a model of normalized current (*I*) based on the circuit to which the piezofilm is attached. Specifically, the normalized current can be replaced by the normalized voltage across the piezofilm divided by the normalized impedance of the total circuit (*I* → *V*_p_/*Z*). Making this substitution in equation (2.7) and solving for *V*_p_ leads to a model for the normalized voltage across the piezofilm

2.9 $$V_{p}=\alpha {\bar{\epsilon }}_{xy}\left(\frac{jZ}{1+jZ}\right)=\alpha Q\hat{\lambda }\frac{h}{\ell }e^{jT}\left(\frac{jZ}{1+jZ}\right),$$

where a coefficient, *α*, has been added as an empirical constant to be fitted. The constant *α* helps to account for unmodelled losses, such as those resulting from the diodes of a voltage bridge or the vortex-induced vibrations of the piezofilm. Note that in the open-circuit case (*Z* → ∞), the voltage follows the strain in both magnitude and phase.

In this study, we will consider a simple charging circuit, where the normalized impedance is the sum of the normalized internal impedance of the piezofilm, *Z*_p_, and the normalized impedance of a charging element, *Z*_c_. The transfer function from normalized voltage across the piezofilm to normalized voltage across the charging element is *Z*_c_/*Z*. The model therefore predicts a normalized voltage across the charging element of

2.10 $$V_{c}=L^{-1}\left[L\left[\alpha Q\hat{\lambda }\frac{h}{\ell }e^{jT}\left(\frac{jZ}{1+jZ}\right)\right]\left(\frac{Z_{c}}{Z}\right)\right],$$

where L and $L^{-1}$ denote the Laplace and inverse Laplace transforms.

We modelled the charging element as a capacitor (normalized capacitance *C*) and resistor (normalized resistance *R*_2_) in parallel (*Z*_c_ = *R*_2_/(*R*_2_ + *SCR*_2_)), where *S* is the complex frequency parameter divided by *ω*), and the total normalized impedance as the normalized impedance of the charging element plus the normalized internal resistance of the piezofilm (*Z* = *Z*_c_ + *R*_1_). Making these substitutions into equation (2.10) leads to

2.11 $$V_{c}=\alpha Q\hat{\lambda }\frac{h}{\ell }\frac{R_{2}(R_{1}+R_{2})}{2(1+{\left(R_{1}+R_{2}\right)}^{2})}\left[K_{1}\sin(2T+\phi_{1})+1-K_{2}e^{-T\frac{1+R_{1}(R_{1}+R_{2})}{R_{2}C(R_{1}^{2}+1)}}\sin\left(\frac{T}{C(1+R_{1}^{2})}+\phi_{2}\right)\right],$$

where *K*_1_, *K*_2_, *ϕ*_1_ and *ϕ*_2_ are constant coefficients that include *R*_1_, *R*_2_ and *C* (see electronic supplementary material). The term scaled by *K*_1_ models a fluctuating component of the capacitor’s voltage due to the oscillating piezofilm, and the term scaled by *K*_2_ models the energy accumulating on the capacitor ($e_{c}=(1/2)cv_{c}^{2}$). Equation (2.11) can be used, for example, to estimate the initial ‘harvesting rate’ by calculating d*e*_c_/d*t*|_*t*=0_ with *K*_1_ = 0.

## 3. Experimental methods

**Figure 1.**
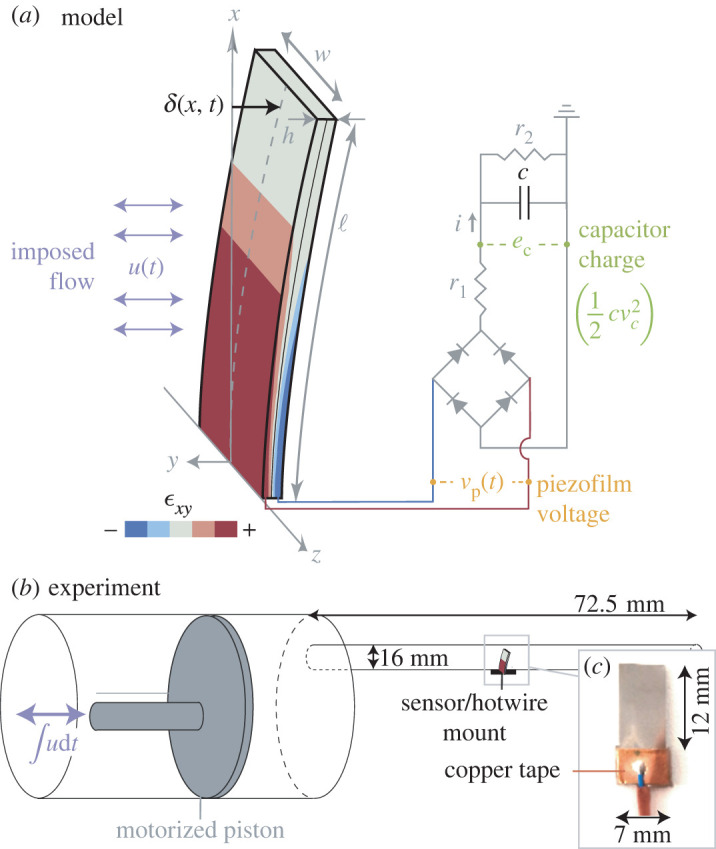
Model/experiment set-up. (*a*) An oscillating flow causes an oscillating strain field (*ε*_*xy*_) and therefore an oscillating voltage (*v*_p_). The AC signal passes through a rectifier, causing charge to accumulate on the capacitor (*e*_c_). (*b*) A motorized piston creates oscillating airflows that pass over a piezofilm or hotwire. (*c*) Photograph of a PVDF piezofilm supported by copper tape.

To test our model, we built a rig that exposes a rectangular piezofilm to oscillating airflows (figure 1). The rig is driven by a lung flow simulator (Hans Rudolph Series 1120) that outputs sinusoidal airflows inspired by human breathing: 197 waveforms with frequencies ranging from 0.13 to 1.67 Hz and amplitudes ranging from 0.1 to 7 l. The airflows pass through an acrylic tube with a 15.6 mm inner diameter, comparable to that of a typical human airway (tracheal diameter approx. 15 mm in a young adult [27]) (figure 1*b*). To avoid entrance/exit effects, the piezofilm was installed more than 10 diameters from either end of the tube. We recorded the turbulence intensity of the flow using a hotwire (Dantec 9055H0211; 4 × 0.235 mm) placed in the centre of the acrylic tube and sampled at 10 kHz.

For the piezofilm, we chose a 28 μm thick PVDF piezoelectric film, which we cut from a bulk off-the-shelf sheet (Component Distributors Inc., 1-1003702-7, bimorph). Thinner material produces larger deflections at low speeds, and PVDF material has a biocompatibility advantage over materials like lead zirconate titanate (PZT) [28]. Our proof-of-concept sensor was the largest possible size for the airway-inspired test section, with dimensions of 7 by 12 mm. Beam theory gives the first resonant frequency of a beam this size as approximately 44 Hz, well above typical breathing frequencies. To measure the voltage across the piezofilm, we placed copper tape at the base of each face and soldered a lead wire to the tape (figure 1*c*). The differential voltage across the leads, *v*_p_, fed into a data acquisition card (National Instruments PCIe-6363) controlled by a custom user interface (LabView 2018).

The voltage across the piezofilm also fed through a full wave rectifier, and the rectified signal charged a 1 μF capacitor for 20 s during each trial. The same data acquisition card also recorded the voltage across the capacitor, *v*_c_. The experimental ‘harvesting rate’ was determined by taking the average slope of the capacitor energy curve over the timespan of the trial ($\bar{{\dot{e}}_{c}}$). To ensure that the capacitor started fully discharged, an N-channel metal-oxide-semiconductor field-effect transistor (MOSFET) switch grounded the capacitor for 5 s before each trial. Each trial was repeated five times to estimate the mean and variability of voltages and harvesting rates.

To test whether sub-resonant forcing could actually run a SPD system (where harvesting rate maps to flow conditions), we built a scaled-up version of our set-up that triggered wireless transmissions (pings). In this set-up, five piezofilms (20 mm × 12 mm × 28 μm) were connected in parallel. Beam theory gives the first resonant frequency of beams this size as approximately 21 Hz, well above typical breathing frequencies. The accumulating charge on two capacitors (5 μF) was fed into an ultra-low-power load switch (Semtech TS12001, operating supply current 70 nA). When the voltage of the storage element exceeded the switch’s internally regulated threshold (*v*_t_ = 2.1 V), a low-power oscillator (SiTime, SiT1533, 32.768 kHz) transmitted a wireless radio frequency (RF) ping. The pings were received and recorded by a software-defined radio (SDR) from SDRplay (RSP2pro). See Lopez Ruiz *et al*. for additional details [29]. In a functioning SPD system, the ping rate would then be mapped to a flow variable of interest, e.g. breaths per minute. This scaled-up prototype is too large for medical applications, but we used it as a proof of concept, i.e. a test of whether our physical model could inform the interpretation of a harvesting-driven ping rate.

## 4. Results

### 4.1. Voltages and harvesting rates depend on flow conditions

**Figure 2.**
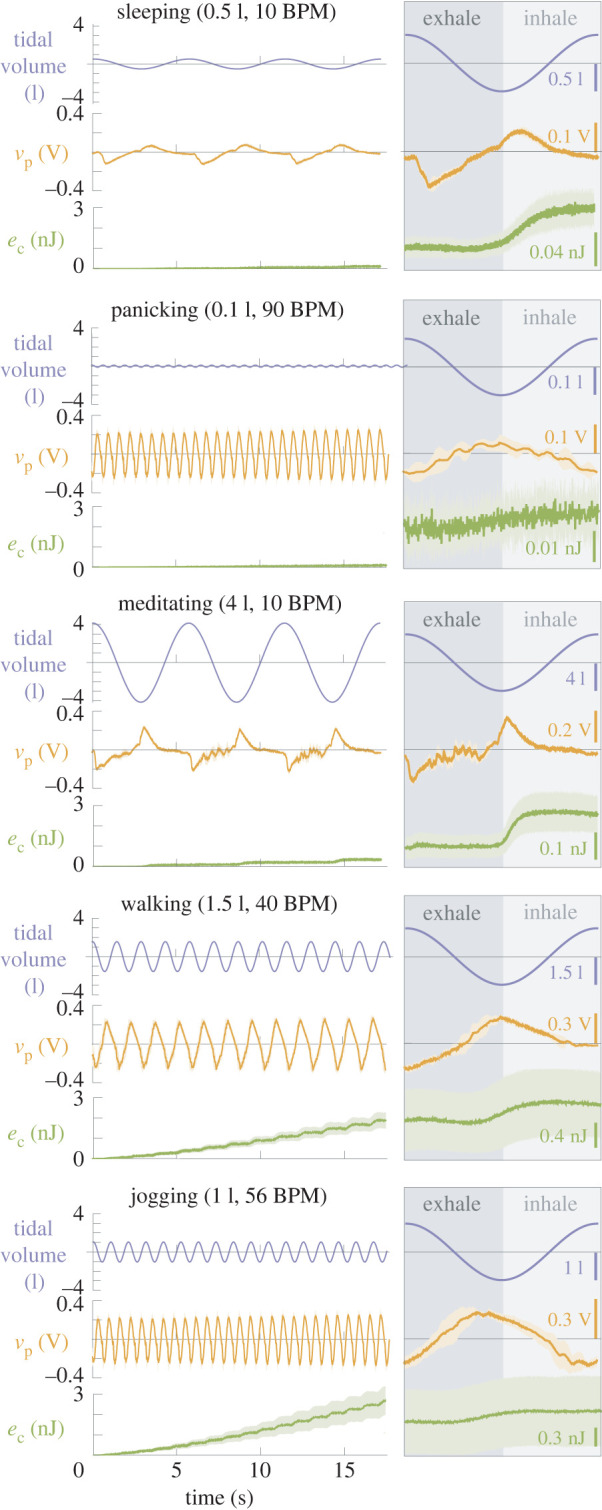
Piezofilm voltage and capacitor charge for five sample breath types. The fluctuating tidal volume (deviation from the resting volume of the lung-flow simulator) of the imposed breaths is shown for reference. Solid curves and shaded bands show mean and mean ±*σ* based on five trials. Zoomed inserts show data for the final breath only.

As expected, the system’s response is heavily dependent on the imposed flow. Consider, for example, five flows that loosely align with breathing amplitudes/frequencies of sleep, meditation, fast walking, jogging and panic [30]. We chose to highlight these breath types because they are recognizable benchmarks that cover a range of frequencies and amplitudes. They also offer interesting test cases because they include cases with similar amplitudes but different frequencies (sleep, panic) and vice versa (sleep, meditation). The voltage across the piezofilm (*v*_p_) responds differently in each of the five flows (figure 2). The narrow ±*σ* bands reveal that the subtle differences in the voltage signals are consistent and significant. For reference, the open-circuit voltages were about four times higher, with peak-to-peak amplitudes ranging up to approximately 2 V.

The voltage signals (*v*_p_) differ between breath types in multiple ways. The clearest change is in the frequency: the dominant frequency of the voltage signal matches that of the imposed breath. A Fourier transform or peak-counting algorithm applied to the voltage signal would identify the breath frequency with high accuracy. The amplitude and shape of the voltage signal are more complex functions of the imposed breath’s amplitude, but our results suggest that a classifier trained on known breath data should be able to differentiate between breath amplitudes. Therefore, a *powered* sensor with a sampling frequency well above twice the breathing frequency (Nyquist criterion) should be able to differentiate breath types based on the voltage signal. However, we will not explore such a classifier, because our goal here is to explore whether the sensor in our system could be SPD, i.e. distinguish breath types based solely on harvesting rate.

**Figure 3.**
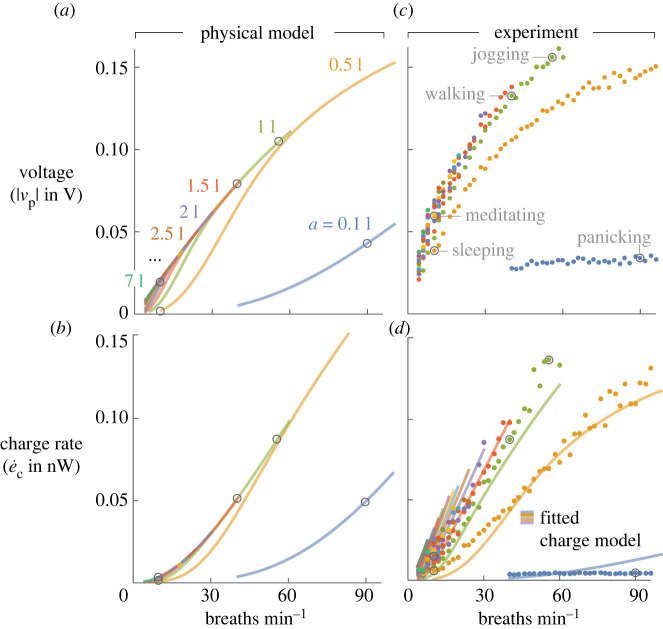
Voltage magnitudes and harvesting rates from experiments and model. (*a*,*b*) Model. Colours indicate breath amplitude in l. (*c*,*d*) Experiments. Solid circles: raw data; solid lines: fitted charge model, i.e. modelled charge rates multiplied by empirically fitted power law (*q*^0.3^*ω*^−1.1^). Five sample breath types from figure 2 located as indicated. Subpanel *d* reprinted with permission from [29].

While the imposed flows did cause a range of harvesting rates (${\dot{e}}_{c}$), many mapped to similar harvesting rates. For example, beyond a certain breath amplitude (approx. 1.5 l), harvesting rates clustered in a narrow band (approx. 0.02−0.05 nW; figure 3*a*,*c*). The ‘sleep’ and ‘panic’ breaths, despite having very different forms, caused about the same harvesting rate, so they could not be distinguished with an SPD implementation of our sensor. Other breath types led to significant differences. The jogging-type flow (high amplitude, high frequency; ${\dot{e}}_{c}=0.128\;\mathrm{nW}$) produced a harvesting rate 25 times that of the sleeping-type flow (low amplitude, low frequency; ${\dot{e}}_{c}=0.005\;\mathrm{nW}$).

To test the scaling predictions of the model, we evaluated equations (2.9) and (2.11) for each of the imposed breath types and with physical constants matching those of our set-up (see electronic supplementary material). The model captures many of the features of the experimental data (figure 3). For example, the model predicts the rise in *v*_p_ with increasing frequency, and it predicts the decreasing slope of the voltage curves, i.e. that increasing frequency has a decreasing marginal benefit on voltage output. It also predicts the collapse of the harvesting rate curves at high amplitudes (figure 3*d*) and that the 0.1 l amplitude breaths—despite reaching the highest frequencies—have some of the lowest voltages and harvesting rates.

These effects can be explained in physical terms thanks to the model. As frequency increases at a fixed amplitude, higher airspeeds impose higher stresses, which lead to higher voltages across the electrodes (equation (2.9)). As for the decreasing slopes of the voltage curves, they are a result of the deflection cap we used in our model (equation (2.5)). Without this cap, the model predicts increasing slopes. The importance of the deflection cap highlights the fact that the sensors are experiencing their maximum deflection at the higher frequencies we measured. As for the collapse of the higher amplitude curves, higher amplitude causes deflection to saturate; further increases have little effect on voltage, and the lower required frequencies lead to more leaked charge per breath. At low amplitudes, the deflections are small, leading to low harvesting rates at all frequencies. Breath types at intermediate amplitudes, such as the jogging-type breath, produce the most power.

Other features of the experimental data are not captured by the model. The low value of the one fitted coefficient (*α* = 0.01; figure 3) shows that the model significantly overpredicts the absolute magnitude of voltage and harvesting rate. Some overprediction is expected given the assumptions in the model. The model assumes perfect voltage rectification, whereas the real system uses off-the-shelf diodes for the voltage bridge. The model assumes a perfectly symmetrical loading, whereas slight misalignments and leakages in the real system caused a voltage offset in the piezofilm (30 mV registered with no airflow). After applying the empirical fit coefficient (*α*) to the voltage signal, the mean RMS error between modelled and experimental charge rates was 0.026 nW. For some of the amplitude curves, this translates to small errors (less than 5%), but for other amplitudes, errors are much larger (greater than 100%).

The model offers physical explanations for qualitative features of the charge rates, e.g. the *relative* changes in harvesting rate across different amplitudes. However, the RMS errors are too high for some model applications. Therefore, we also tried modifying our physical model by multiplying with an empirically fitted power law model for forcing amplitude and frequency ($q^{\alpha }\omega^{\beta }$, where *α* and *β* are constants), the idea being to factor in some of the nonlinearities of the real system. This additional fitting increased the model’s accuracy, with mean RMS error dropping to 0.008 nW (figure 3*d*). The power law has no physical origin, so it lacks the physics-based nature of the base model, but it offers more accurate results for those wanting to use the model as a predictive tool. Note, however, that the model is only tested here for one sensor geometry in one class of flow environment (sensor perpendicular to sinusoidal flows through a cylinder); how the model scales to other SPD systems remains an open question.

### 4.2. A demo 'self-powered by design' system detects breaths per minute

**Figure 4.**
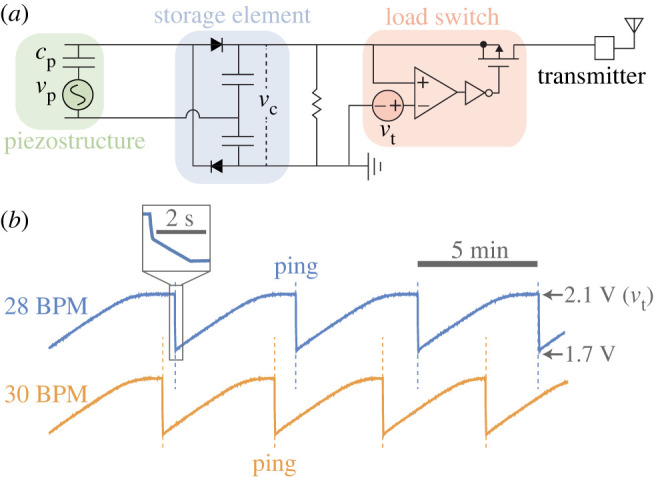
SPD sensing demo. (*a*) Layout of the SPD circuit: the oscillating piezofilm voltage (*v*_p_) charges a storage element. When the capacitor voltage (*v*_c_) exceeds a threshold (*v*_*t*_), a load switch closes and causes a transmitter to ping, which brings the storage element back to its starting voltage (1.7 V). (*b*) The transmitter produces a higher ping rate as breaths per minute (BPM) rises from 28 to 30. Reprinted with permission from [29].

The fact that harvesting rates can distinguish some breath types (figure 3*b*,*d*) implies that SPD sensing is possible in at least some sub-resonant scenarios. Our demo SPD system (five scaled-up versions of the piezofilm; see Experimental methods) demonstrates this concept, albeit on an oversized scale. As breath frequency changes (from 28 to 30 breaths per minute), so too does the time between wireless pings (approx. 300 to 260 s or 140 to 130 breaths) (figure 4*a*). No components of the oscillator circuit were powered; the pings were generated entirely by energy from the airflow. A receiver recording the pings could differentiate between these two breathing rates, despite the sensor having no power source.

According to our model, ping frequency is approximately ${\dot{v}}_{c}/\Delta v_{t}$, where Δ*v*_t_ is the voltage increase needed to trigger a ping. Given the breath conditions from our SPD demo (4 l; 28 BPM →30 BPM), the model predicts a 13% increase in ping rate based on equation (2.11), which is comparable to the increase observed experimentally (figure 4*a*; +17±10%). Modelling ping rate is a key design constraint, because ping rate determines the temporal resolution of SPD sensing. By the Nyquist criterion, changes in flow patterns could only be detected at frequencies less than one-half the ping rate. We only have one test case of the model’s ping rate predictions, but with further validation, the model could theoretically offer guidance for designing a ping-based SPD system.

## 5. Discussion

### 5.1. Power budget considerations

**Figure 5.**
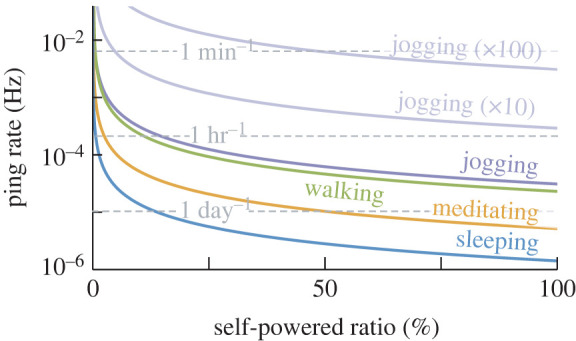
Predicted ping rate depends on breath conditions and self-powered ratio. Coloured lines indicate ping rates based on the predicted harvesting rates for each breath type (figure 3) and the power requirements of our demo SPD system (figure 4). Self-powered ratio denotes the per cent of the energy budget that is self-powered (i.e. not battery-dependent). Translucent lines show theoretical values based on amplified harvesting rates.

Our benchtop SPD system (figure 4) was able to differentiate between two breath frequencies using only energy from the airflow itself. However, based on its ping rate, the system could only detect changes in flow conditions over timescales measured in minutes. These constraints are on an enlarged prototype. Constraints would be tighter still on the prototype sized for a human airway (figure 1*c*). The pings of this smaller prototype would be hours apart in the jogging-inspired flow and days apart in the sleeping-inspired flow (figure 5).

One way to raise the ping rate is to supplement with battery power. For example, if 75% of the energy budget were drawn from a battery (a ‘self-powered ratio’ of 25%), the ping rate could be quadrupled, and ping rates in the jogging-inspired flow could rise closer to 1 h^−1^ (figure 5, jogging). The ping rate would also increase with a lower-power transmission system (our demo SPD system requires 7.3 μW during transmission). Without relaxing the requirement of self-powered operation or changing the transmission circuit, increasing ping rate would require a change in the harvesting rate itself. With a harvesting rate 100-times amplified, the ping rate in the jogging-inspired flow could rise closer to 1 min^−1^ (figure 5, jogging × 100).

One advantage of our model is that it offers a set of strategies for amplifying harvesting rate. Based on equation (2.11) with jogging-inspired forcing, doubling the sensor’s permittivity (*k*) would increase harvesting rate by 15%, and doubling its piezoelectric coefficient (*d*) would increase harvesting rate by 95%. Doubling *d* is well within reach: new single-crystal materials have been demoed with piezoelectric coefficients 200 times higher than PVDF [31]. With better materials, shrinking the cantilever could become an option. In this case, a change in dimension would affect multiple variables, and the model could be used to consider the trade-offs of such coupled effects. For example, holding all else constant, halving piezofilm length (ℓ) would decrease harvesting rate by 50%. In reality, halving length would also roughly halve the forcing (*q*) and the capacitance/resistance of the piezo (*c* and *r*_1_), the net effect of which is an 85% decrease in the modelled charge rate (${\dot{e}}_{c}$).

Note also that we chose a rectangular, full-electrode beam as an archetypal validation case, but other configurations can be more efficient. Partial-electrode configurations can allow higher mean stresses in active elements and lower overall impedance, leading to higher efficiency [22,32]. For example, the model predicts that if the electrodes were affected by only the stresses in the bottom quarter of the beam (averaging from *X* = 0 to *X* = 0.25 instead of *X* = 0 to *X* = 1 in equation (2.6)) harvesting rate would increase by 230%.

### 5.2. Potential applications to pulmonary diagnostics

The ping rate of an SPD sensor would determine the timescales at which the airway could be monitored with no battery. Ping rates on day-to-day scales could offer longitudinal breath diagnostics and perhaps track the long-term progression of a disease. Ping rates on minute-to-minute scales could offer more vigilant sensing options. They could, for example, look for the subtle changes in breath patterns that can occur before an asthma exacerbation [33].

If SPD operation were feasible for a given use case, the resulting sensor could offer a significantly smaller form factor than existing sensing solutions. Typical sensing solutions use a nasal cannula or facemask to route a patient’s breath directly through an anemometer [13–16]. The anemometer may use a thermistor or pressure transducer to correlate temperature/pressure with airspeed. Indirect respiratory sensors correlate breath with secondary physiological effects. Motion of the chest cavity can be measured with accelerometers [12] or resistive bands [34]. Trans-thoracic electrical impedance—which changes as lungs fill with air—can be measured by an impedance pneumograph [35]. Tracheal acoustics can be measured by microphones mounted to a patient’s neck [36]. The fluctuating moisture content of breath can be measured by humidity sensors beneath a patient’s nose [11]. All of these wearable approaches are bulky and cumbersome compared with an implantable solution, and they can disrupt the patterns they seek to measure: nose and mouthpieces can reduce breath frequency and raise tidal volume [37].

Implantable airway sensors have only recently been studied. A 2010 US patent proposed the general concept of implantable airway sensors, mentioning Doppler velocimetry, pressure gauges and particle/chemical detection as potential sensing modalities [38]. Two implantable airway sensors have been tested *in vivo* in research settings. One used ultrasonic transmitters mounted in the trachea [39]; the other used an implantable catheter with two hotfilm sensors [40]. These devices surround the airflow and require major surgery to install. A small SPD sensor could in theory be implanted with less invasive surgery, e.g. through a small incision in the cricothyroid membrane, an airway access point with surgical precedent [41].

### 5.3. Model suggests a method for broadband 'self-powered by design' sensing

Existing SPD approaches are inherently narrowband, because they rely on a single-variable function ($R^{1}\to R^{1}$) mapping duty cycle to some scalar of interest, such as flow rate [19,20,42] or deformation [18]. However, a form of *broadband* SPD sensing can be hypothesized based on our model. Proving this broadband technique to be feasible would require experiments that we leave for future work, but the potential applications are novel enough that we chose to finish this paper with a brief exploration of the hypothesis.

Both the experiments and model confirm that an SPD sensor’s ping rate depends on the frequency content of the surrounding flow (figure 3). This mapping from frequency to ping rate depends on the geometrical and material properties of the sensor. A diverse array of SPD sensors would therefore produce an array of ping rates that contains information about the frequency content of a flow environment. By capturing frequency content, a sensor could theoretically differentiate between non-sinusoidal airflows, which would enable more advanced flow-sensing applications.

**Figure 6.**
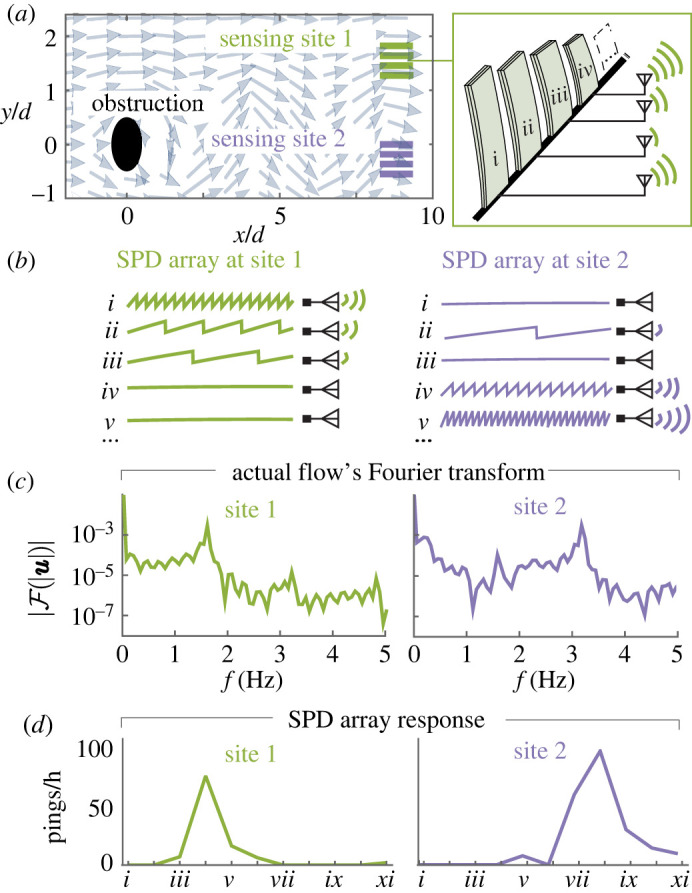
Simulated demo of an SPD array. (*a*) Flow field from a simulated wake downstream of an obstruction [43,44]. Inset: SPD array with piezoelectric beams ranging from 5 to 15 cm in increments of 1 cm. (*b*) A simulated 11-element SPD array responds differently at the two sensing sites in (*a*) (curves show energy by each element according to equation (2.11); energy drops denote pings). (*c*,*d*) The SPD array’s ping rates contain frequency information about the flow, as can be seen by comparing with the flow’s Fourier transform at the two sensing sites.

To explore the implications of broadband SPD sensing, we used a published broadband flow field [43,44] to provide the forcing functions for an array of our SPD sensor models. Specifically, we used the time-varying local flow speed at two sites in the flow field to scale the forcing (*Q* in equation (2.3)) on each element of an 11-element SPD array (figure 6*a*). The resulting array of ping rates is site-dependent figure 6*b*). In an application setting, the SPD sensors could produce pings with a unique width so that the ping rates could be mapped to their respective sensors by a single receiver. Because the ping rates are frequency-dependent, they contain information—when analysed together—about the Fourier transform (frequency content) of the flow at each site (figure 6*c*,*d*).

The turbulence intensity we measured via hotwire offers some evidence that this broadband sensing concept could work experimentally. The ‘turbulence intensity’ of the flow is $u^{\prime }/\bar{u}$, where $\bar{u}$ is average signal and *u*′ is the root mean square of the signal after the average is subtracted. The ‘fluctuation intensity’ of the voltage across the piezofilm is the same intensity with voltage in place of *u*. Across the frequencies and amplitudes we tested (figure 3), these two fluctuation intensities are correlated with an *R*^2^ value of 0.71. The correlation suggests that the frequency content of the flow is roughly mapping to frequency content of the sensor’s electrical response, which is critical for the frequency-dependent ping rates that drive the broadband sensing concept.

Broadband SPD sensing could open up new horizons for self-powered sensors. To date, SPD sensors have only monitored one-dimensional input; monitoring frequency spectra would unlock new applications. In the larynx and aorta, turbulence—known for its predictable frequency spectrum [45]—can be a predictor of pulmonary [46,47] and cardiovascular [48] health. Implantable SPD sensor arrays could in theory monitor for dangerous wake signatures the way that predator animals monitor for the wake signatures of their prey [49].

## 6. Conclusion

Battery replacement/recharging is a significant obstacle to widespread acceptance of implantable flow sensors that are not one-time use. In particular, SPD sensing offers a minimalist and batteryless flow sensing solution. Our results show that SPD sensors—at least those made with off-the-shelf piezoelectric materials like PVDF—probably cannot provide airway sensing with better than hour-by-hour resolution in the near future. However, our model offers a framework for working towards SPD implantables. We only tested the model for one sensor geometry in one class of flow environment, but if it were validated in other systems, it could provide a roadmap for future generations of SPD sensors. It could offer a way to explore (i) what applications are possible given available materials and size constraints and (ii) what input variables to consider when designing SPD sensors for those applications.

## Data Availability

All data can be found within the manuscript or within the electronic supplementary material [50].
